# Electrochemical Performance of Symmetric Solid-State Supercapacitors Based on Carbon Xerogel Electrodes and Solid Polymer Electrolytes

**DOI:** 10.3390/gels9120983

**Published:** 2023-12-15

**Authors:** Boryana Karamanova, Emiliya Mladenova, Minju Thomas, Natalia Rey-Raap, Ana Arenillas, Francesco Lufrano, Antonia Stoyanova

**Affiliations:** 1 Institute of Electrochemistry and Energy Systems, Bulgarian Academy of Sciences, 1113 Sofia, Bulgaria; boriana.karamanova@iees.bas.bg (B.K.); e_mladenova@iees.bas.bg (E.M.); 2 CNR-ITAE, Istituto di Tecnologie Avanzate per l’Energia “Nicola Giordano”, 98126 Messina, Italy; minju.thomas@itae.cnr.it (M.T.); francesco.lufrano@itae.cnr.it (F.L.); 3 Instituto de Ciencia y Tecnología del Carbono, INCAR-CSIC, Francisco Pintado Fe, 26, 33011 Oviedo, Spain; nataliarey@uniovi.es (N.R.-R.); aapuente@incar.csic.es (A.A.)

**Keywords:** carbon xerogel, activated carbon YP-50F, Aquivion electrolyte membrane, solid-state supercapacitor, long lifetime

## Abstract

For the development and optimization of solid-state symmetrical supercapacitors, herein, we propose using carbon-based electrodes and sodium- and lithium-form Aquivion electrolyte membranes, which serve as the separator and electrolyte. Carbon xerogels, synthesized using microwave-assisted sol-gel methodology, with designed and controlled properties were obtained as electrode materials. Commercial activated carbon (YP-50F, “Kuraray Europe” GmbH) was used as the active material for comparison. Notably, the developed solid-state symmetrical supercapacitors provide sufficiently high specific capacitances of 105–110 F g^−1^ at 0.2 A g^−1^, along with an energy density of 4.5 Wh kg^−1^ at 300 W kg^−1^, and a voltage window of 0–1.2 V in aqueous environments, also demonstrating an excellent cycling stability for up to 10,000 charge/discharge cycles. These results can demonstrate the potential applications of carbon xerogel as the active electrode material and cation exchange membrane as the electrolyte in the development of solid-state supercapacitor devices.

## 1. Introduction

Supercapacitors (SCs) bridge the gap between traditional dielectric capacitors and batteries by providing several orders of magnitude higher energy density than dielectric capacitors and possessing a higher power density and longer cycle life than batteries [1]. Flexible energy storage devices such as solid-state supercapacitors are becoming the most attractive electrochemical energy systems due to their flexibility, wear resistance, mechanical properties, low weight, no electrolyte leakage, etc. [2]. However, the flexibility and safety of supercapacitors have become key factors that place higher demands on researchers, and developing such devices with better electrochemical performance is still a huge challenge for them [3].

Each component of a supercapacitor (separator, electrode, and electrolyte) has a significant influence on its electrochemical performance [4]. The properties of electrode materials and their physical structures directly determine the overall capacitive performance of this system [5]. This requires that the electrode material has a larger specific surface area and is in contact with the electrolyte to the greatest extent possible to improve the electrochemical efficiency of the device [6].

The effect of the interaction between the electrodes and the electrolyte is also an important factor, and understanding the interactions between them is essential in designing more efficient electrode materials. In addition, it is known that the electrochemical behaviors of the electric double-layer electrodes are determined not only by the exposed surface area of the carbon electrode but also by the matching degree between the pore size distribution of the carbon electrode and the size of solvated ions in the electrolytes [7,8].

Various porous carbon materials are widely used as electrode materials in supercapacitor systems, and carbon aerogels and xerogels are considered promising alternatives due to their good properties, such as electrical conductivity, high specific surface area, three-dimensional porous structure, and the ability to control the pore size distribution.

Carbon gels are promising materials for energy applications due to a number of their interesting characteristics, such as the unique three-dimensional nano-lattice, porous structure, high electrical conductivity, and controlled properties [9]. The specific surface area of organic xerogels is usually low (about 200 m^2^ g^−1^), but its value can increase up to 600–700 m^2^ g^−1^ after the pyrolysis step under certain operating conditions [9,10]. Through chemical activation, the microporosity of the material can be further increased to surface area values of almost 2000 m^2^ g^−1^ [11,12]. Many factors are involved in this process, which makes it possible to design the porosity of carbon xerogels by selecting specific activation parameters, such as the activation agent and precursor used, activation temperature and time, etc. [12,13]. All these indicate the great potential for obtaining carbon xerogels with controlled micro/mesopores and the influence of synthesis conditions on their energy storage capacity.

Numerous studies have been conducted on supercapacitors using aqueous electrolytes based on lithium or sodium sulfate [14,15]. The choice of the cation has been determined by investigating sulfate salts with different alkali metal cations (such as Li^+^, Na^+^, and K^+^) and their influence on the electrochemical characteristics of these systems [16,17]. An advantage is that sulfates can be applied not only as liquid state electrolytes but also as gel electrolytes [18]. The economic aspect is also very important, considering that Li_2_SO_4_ and Na_2_SO_4_ are the cheapest among the alkali metal sulfate salts [19].

The best results obtained for an aqueous solution of Li_2_SO_4_ are explained by the ion sizes. In aqueous solution, the alkali metal ions are highly dissolved and the diameter of the ion-solvent complex increases in the order K^+^ < Na^+^ < Li^+^. The highest capacitance values obtained for the largest and most dissolved ions are due to their low mobility and low diffusion coefficient. Na^+^ and K^+^ cations are characterized by a smaller ion–solvent complex diameter and are have higher mobilities and diffusion coefficients, along with their solvation/desolvation energy; therefore, they can be sorbed differently in the micropores [16]. The result obtained can be explained by the fact that in an electric double-layer capacitor (EDLC), very high ion mobility is not required for charging and discharging the double electric layer, in contrast to pseudocapacitive systems where fast redox processes require fast ion transfer at the electrode/electrolyte interface [16].

The research and development of flexible solid polymer electrolyte membranes (PEMs) and anion exchange membranes (AEMs) have been subject to increasing interest in recent years. Among polymer electrolyte membranes, the best known are per-fluorinated sulfonic acid (PFSA) ionomers, including Nafion and Aquivion, which are frequently used in electrochemical applications such as fuel cells, water electrolysis, and supercapacitors [20]. PFSAs possess a high ionic conductivity, good mechanical stability, and excellent chemical stability to ensure the effective and stable operation of flexible solid-state supercapacitors [21]. Among the wide range of PFSA ionomer membranes, along with Nafion, Aquivion^®^E87-05S has a growing role in these applications. This membrane shows higher proton conductivity and thermal stability than Nafion due to its shorter side chain, higher crystallinity, higher glass transition temperature, and lower equivalent weight [22,23]. Aquivion membranes are also chemically stable and can operate at higher temperatures than Nafion [24]. 

The Na^+^ exchange Aquivion membrane has been successfully used as an electrolyte membrane in supercapacitors, demonstrating superior performance for more than 15,000 cycles and 210 h of floating at 1.6 V, without noticeable degradation of the electrochemical properties and exceeding those of the Nafion membrane [25,26]. Our previous study found that the symmetric supercapacitor with the K^+^–form Aquivion membrane as the electrolyte and separator showed very stable capacitance performance [27].

In the development of supercapacitors, it should also be foreseen that the selection of separators with better performance can have a decisive impact on the integration of their overall performance [28].

The aim of this work is the synthesis of nano-structured carbon xerogels using the microwave-assisted sol-gel methodology with designed and controlled properties and their use in the electrode preparation and fabrication of symmetric solid-state supercapacitors. For their optimization, it is proposed to use the Na^+^ and Li^+^ exchange Aquivion^®^ E87-05S electrolyte membrane, which serves as the separator and electrolyte. The assessments of the electrochemical characteristics were performed using cyclic voltammetry (CV), galvanostatic charge/discharge measurements, electrochemical impedance spectroscopy (EIS), and long-term durability tests. The capacitance performances of the obtained carbon xerogels were compared to those of a commercial activated carbon (YP-50F, “Kuraray Europe” GmbH) in order to establish the structural and surface characteristics of carbon materials that may influence the performance of solid-state supercapacitors.

## 2. Results and Discussion 

### 2.1. Physical, Chemical, and Morphological Characterizations of Carbons 

The activated carbon xerogel obtained is a synthetic carbon with no impurities and a low content of oxygen. Table 1 shows the elemental analysis of the activated carbon xerogel (AX) and the commercial carbon used for comparative purposes. As can be observed, the chemical composition of both carbons is very similar, mainly composed of carbon.

**Figure 1:**
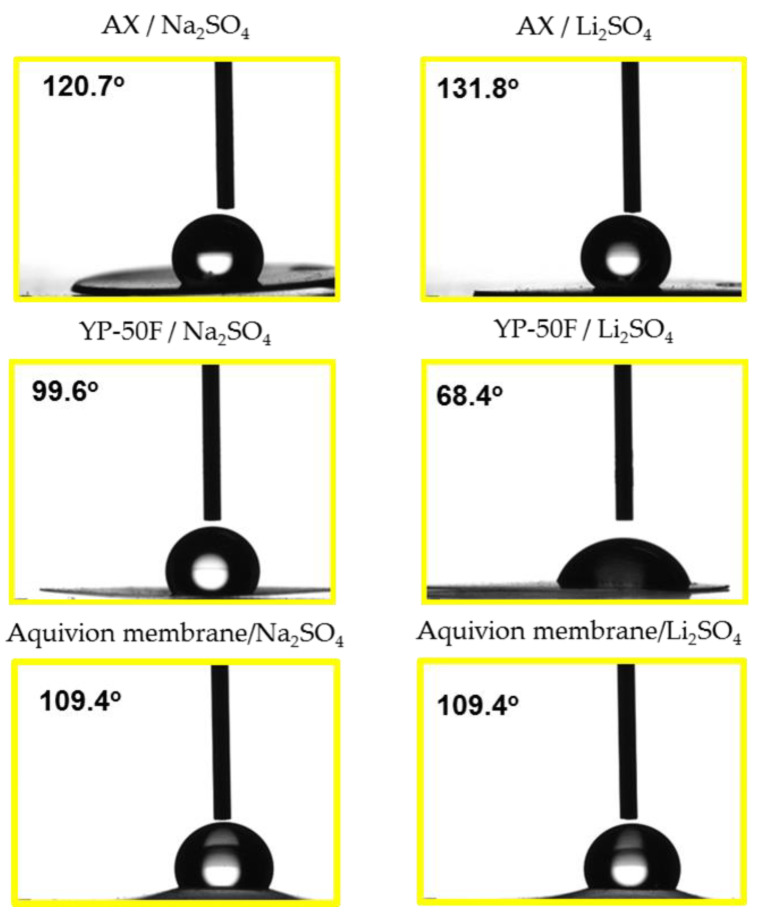
Electrode wetting angles of the electrodes made of AX and YP-50F, in addition to the Aquivion membrane versus Na_2_SO_4_ and Li_2_SO_4_ (both 1 M).

Although the two carbons did not differ substantially from a chemical point of view, the different nature of the carbons may influence their further interactions with electrolytes. There is not a specific measurement to analyze such interactions, but the wetting angle could be used to evaluate the interaction of the electrodes surface and the electrolytes evaluated. Therefore, the wettability of AX and YP-50F, as part of their electrodes, versus Na_2_SO_4_ and Li_2_SO_4_ electrolytes were measured and the results are shown in Figure 1.

It was observed that whilst the Aquivion membrane shows no differences in wettability in response to both electrolytes (1 M Na_2_SO_4_ and 1 M Li_2_SO_4_), there are clear differences in the wettability of the carbons. The activated carbon xerogel exhibited better wettability with Na_2_SO_4_ than with Li_2_SO_4_, whilst YP-50F shows the opposite trend, with a clear affinity for Li_2_SO_4_. This could be very relevant in their further electrochemical behaviors in the cell.

**Figure 2:**
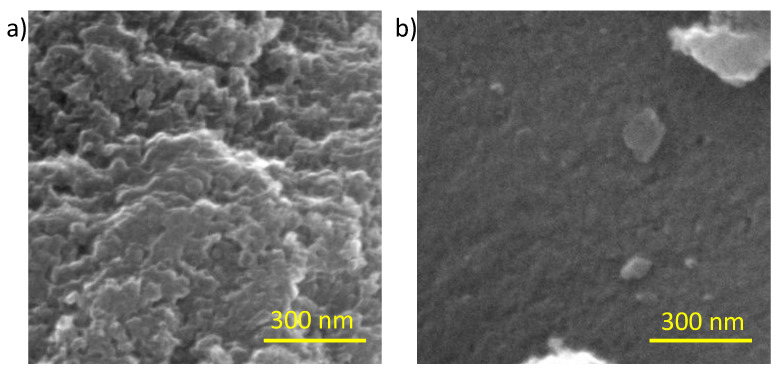
SEM photographs of carbon AX obtained using a sol-gel process in this work (**a**) and the commercial carbon YP-50F (**b**).

Both carbons were also characterized using SEM analysis, and Figure 2 also shows a clear difference in the surface morphology. 

As can be observed in Figure 2, the morphology at the nanoscale is also very different for both samples. The AX shows higher surface roughness and some clear feeder pores are observed. On the contrary, YP-50F shows no roughness and the present porosity should be narrower than in the activated carbon xerogel. 

**Figure 3:**
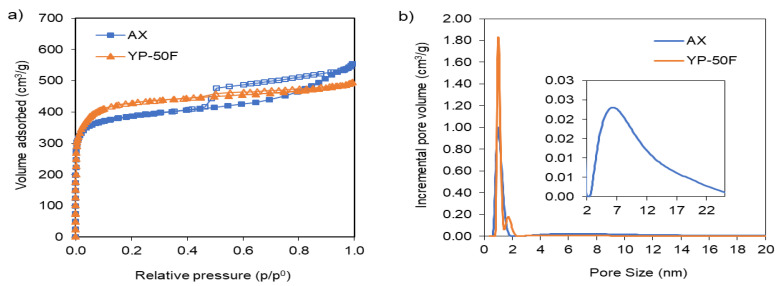
N_2_ adsorption-desorption isotherms (**a**) for the carbon samples studied and their pore size distributions (**b**) according to the NLDFT model.

In order to analyze the porosity of both samples, N_2_ adsorption-desorption isotherms were performed. Isotherms are presented in Figure 3 and the main porosity parameters are summarized in Table 2.

Both carbons have high volume of microporosity, as can be observed in Table 2. The microporosity is quite close between samples, although the commercial YP-50F has slightly higher micropore volume and therefore a higher specific surface area. The total volume of pores (V_t_) is also very close between both samples. However, when observing the N_2_ isotherms, shown in Figure 3, the shape is a bit different, indicating a different porous structure, as was previously expected from the SEM micrographs. The YP-50F isotherm is type I, which is characteristic of microporous samples, with a high increase in the volume adsorbed at low relative pressures and then no increase in the adsorption. On the other hand, the activated carbon xerogel AX presents an increase in the volume adsorbed at low relative pressures, indicating the presence of microporosity, but then there is a constant increase in the volume adsorbed in the whole range of pressures with the typical presence of a hysteresis loop, indicating the existence of mesoporosity. Applying the non-local density functional theory (NLDFT) method to the adsorption data, a pore size distribution can be obtained (Figure 3b). It can be observed that YP-50F is just a microporous sample, with pores < 2 nm exclusively, whilst AX has micropores and feeder pores centered at 10 nm, as was expected from the sol-gel synthesis conditions.

Therefore, although the chemical analysis is very similar between both samples (AX and YP-50F), some differences in wettability with the electrolytes were detected. On the other hand, although both samples present microporosity and very close surface area values, it is clear that different porous structures are present in both samples. The presence of feeder pores in the case of AX may influence diffusional aspects; in fact, the external surface area is quite higher in the case of the AX sample. This could influence in the electrode conformation and the further behavior in the supercapacitor cell. 

### 2.2. Electrochemical Characterization of Symmetric Supercapacitors

Several methods have been applied to study the electrochemical characteristics of activated carbon xerogel (AX) more precisely. The supercapacitor cells were investigated under identical experimental conditions, allowing a comparison with commercial carbon (YP-50F) as well as between the two solid electrolytes (Na^+^ and Li^+^ exchange Aquivion membrane). To follow the changes that occurred in the electrode, CV and EIS curves were recorded at the beginning of the experiments and after performing galvanostatic charges/discharges at different current densities (100 to 1000 mAg^−1^) and a long cycle test (10,000 cycles at 200 mAg^−1^).

**Figure 4:**
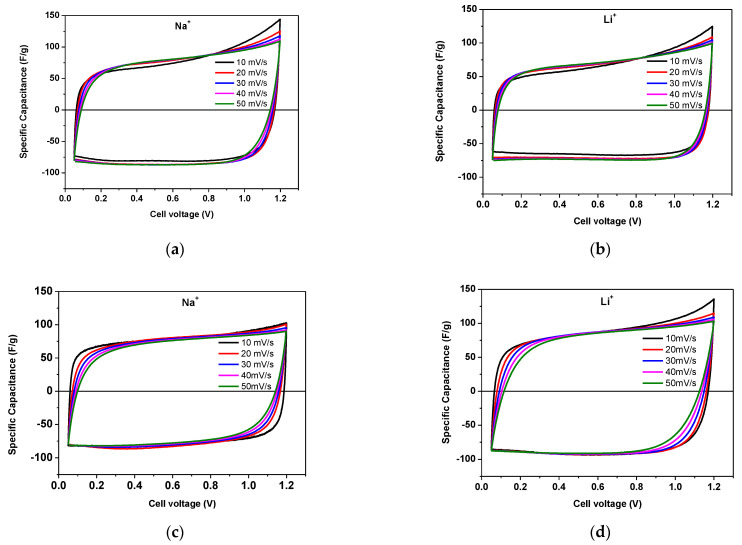
Cyclic voltammogram carried out from 10 to 50 mVs ^−1^ in the voltage range from 0.05 to 1.2 V of symmetric supercapacitors based on AX (**a**,**b**) and YP-50F (**c**,**d**) electrodes and using Na^+^– and Li^+^–form Aquivion membranes.

Figure 4 compares the voltammograms of AX and YP-50F cycling in lithium- and sodium-based polymer electrolyte membranes. In general, all voltammograms are typical for symmetric supercapacitor systems with almost rectangular shapes that almost overlap each other when the scan rate is increased from 10 to 50 mVs^−1^. The rectangular shape of the voltammograms is maintained without any distortion even at a high scan rate, suggesting the excellent capacitive behavior of the devices [29]. 

**Figure 5:**
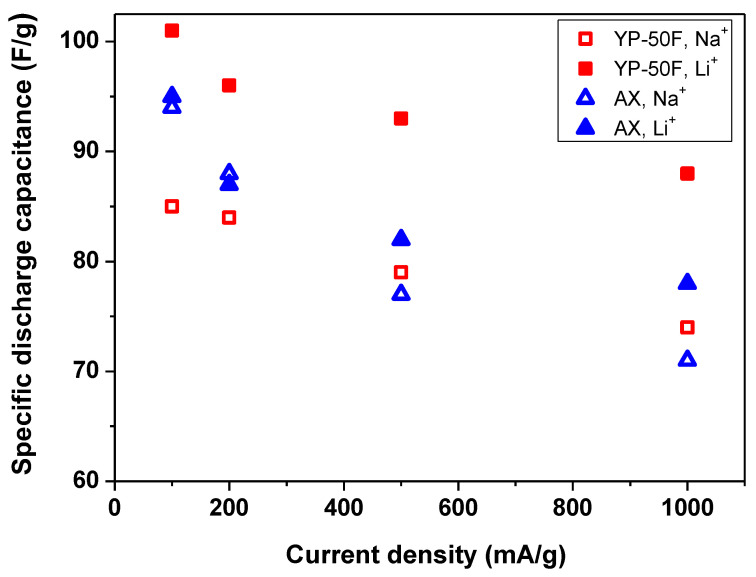
Capacitance trend as a function of current density for different symmetric supercapacitors based on AX and YP-50F electrodes and using Aquivion electrolyte membranes.

To further distinguish the characteristics of AX and YP-50F in lithium and sodium electrolytes, galvanostatic charge/discharge tests were conducted. For a better comparison, their calculated discharge capacitances (according to Equation (2)) and their dependence on the discharge current density are compared in Figure 5. 

There are several points that need to be outlined. The specific capacitance decreases with increasing current density, probably due to the remarkable enhancement of the diffusion limitation inside the deeper pores [30]. This capacitance decline is most pronounced for a cell with activated carbon xerogel (AX) and Na^+^ exchange membrane. The comparison of the galvanostatic charge and discharge (GCD) curves reveals also that the capacitances are higher in both cases for the Li^+^ electrolyte than Na^+^. This result correlates well with studies in aqueous solutions, according to which the better performance of Li_2_SO_4_ compared to Na_2_SO_4_ and K_2_SO_4_ in symmetric supercapacitors is due to their low mobility and low diffusion coefficient (charge/discharge of EDLC does not require very high ion mobility) [24,31]. Electrochemical tests conducted in 6 M NaOH, KOH and LiOH alkaline electrolytes on two active carbons (YP-50F and YP-80F) show that their capacitive characteristics are a consequence of both the different conductivities of the electrolyte and the interaction between the electrolyte and the functional groups of the electrode material. For example, the capacitance of YP-50F was found to improve in the following order: NaOH < LiOH < KOH. Meanwhile, for YP-80F the order changed to LiOH < NaOH < KOH [32].

On the other hand, the results in Figure 5 show that YP-50F has a better initial capacitance than AX, and this result may be related to the different pore textures and wetting angle mentioned above. It is found that the pore size distribution of carbon materials can affect the access of electrolyte ions to the electrode surface, which influences the capacitance and rate capability of the system [33]. Zhao et al. suggested that the effects of surface wettability affect the thermodynamic and dynamic properties, and it can be improved by controlling the surface functional groups of the carbon material [34].

YP-50F shows much better wettability than the AX carbon xerogel, especially in Li_2_SO_4_ (Figure 1), and therefore better surface wettability is obtained with this electrolyte, which is one of the possible reasons for the obtained highest initial capacitance. On the other hand, the different surface morphologies of the two carbons determine their different capacitive behaviors. To explain the results obtained, it should also be kept in mind that YP-50F is only a microporous sample with pores < 2 nm, and this means that all pores of YP-50F are likely accessible to the alkali ions. AX has micropores and feed pores and therefore a much larger external surface area, which affects ion diffusion.

### 2.3. Long-Term Durability of the Investigated Supercapacitors

**Figure 6:**
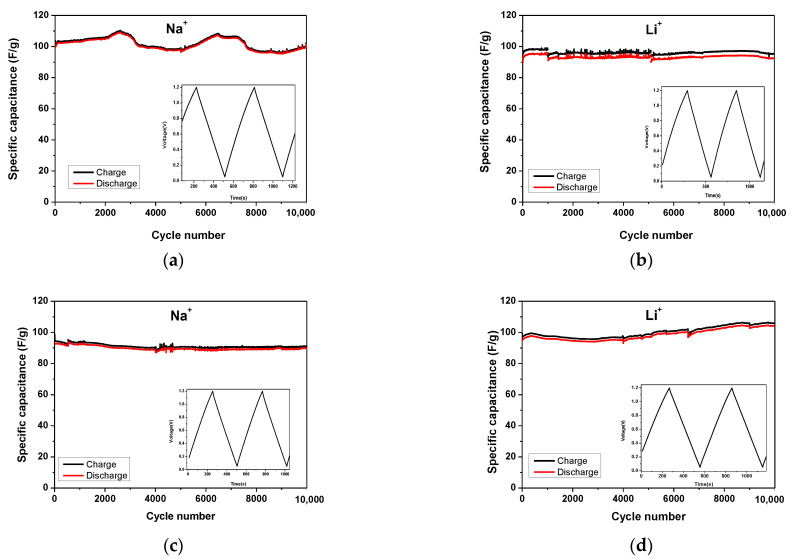
Long-term tests at 200 mAg^−1^ of symmetric supercapacitors with AX (**a**,**b**) and YP-50F (**c**,**d**) using Na^+^– and Li^+^–form Aquivion membranes. The inset is the typical GCD profile.

The characteristics of carbon electrodes with Na^+^– and Li^+^–form Aquivion membranes were further studied based on their cycling stability (Figure 6). Long-term testing includes 10,000 charge and discharge cycles at a current load of 200 mAg^−1^. In general, AX and YP-50F demonstrate excellent cycling stability in both electrolytes (less than 3–4% of variability in specific capacitance after 10,000 cycles). These results are well shown from the high Coulombic efficiencies reported in Table 3. A low initial efficiency of the capacitor based on AX and the Li^+^ Aquivion membrane is ascribed at low wettability of the carbon. This probably means that the ionic conductivity of the electrolyte and the ionic sizes of Li^+^ and Na^+^ are not the only factors that contribute to the performance of the supercapacitor [30].

**Figure 7:**
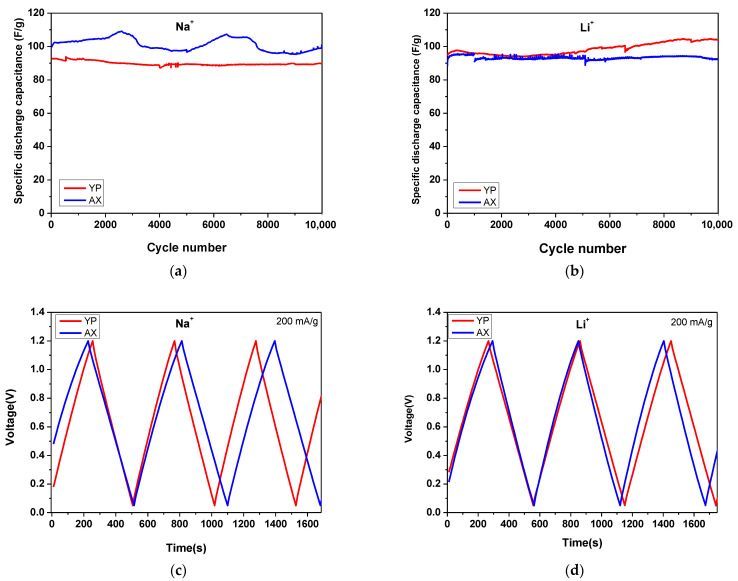
Long-term GCD durability tests (**a**,**b**) and GCD profiles (**c**,**d**) at 200 mAg^−1^ for symmetric supercapacitors with AX and YP-50F using Na^+^– and Li^+^–form Aquivion electrolyte membranes.

After the completing 10,000 cycles, the best electrochemical performance is observed for YP-50F electrodes operating in Li^+^ electrolyte, with a capacitance of 105 Fg^−1^ and a Coulombic efficiency over 98% (Table 3 and Figure 7). It can also be found that the AX sample exhibits higher capacitance than the YP-50F in the Na^+^ electrolyte at 99 Fg^−1^ vs. 89 Fg^−1^ after 10,000 charge–discharge cycles. This result corresponds well with that shown in Figure 5. A possible reason for this is the dominant influence of the conductivity of the Na^+^ electrolyte, as well as the change in wettability and interactions between it and the carbon electrode during the process of cycling stability.

**Figure 8:**
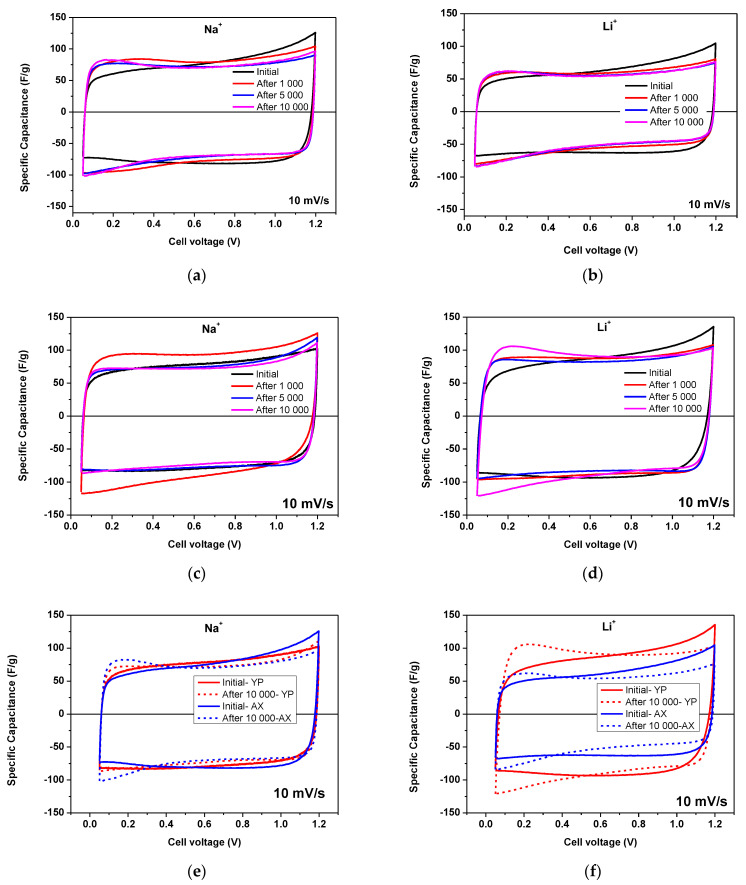
Cyclic voltammogram carried out at 10 mVs^−1^ in the voltage range from 0.05 to 1.2 V using Na^+^– and Li^+^–form Aquivion membranes with AX (**a**,**b**) and YP-50F (**c**,**d**) after 1000, 5000 and 10,000 cycles. Comparative study with the same electrolyte (**e**,**f**).

After 1000, 5000, and 10,000 galvanostatic charge/discharge cycles, CV curves were recorded for each supercapacitor, as shown in Figure 8, at 10 mVs^−1^. As can be seen, the rectangular shapes for all supercapacitors remain preserved, with a slight area shrinkage observed. This result confirms the very excellent stability of these designed solid-state supercapacitors.

**Figure 9:**
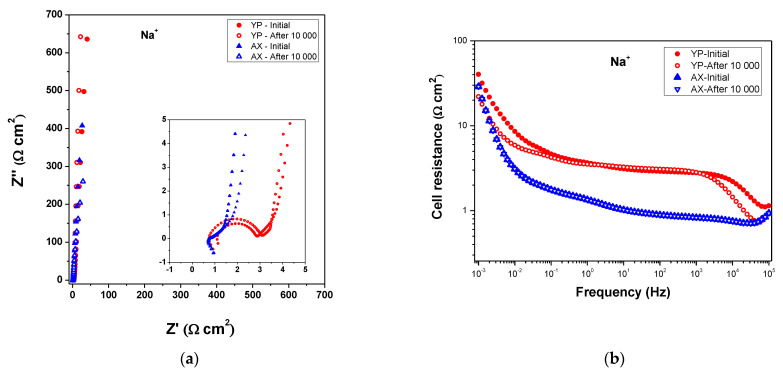

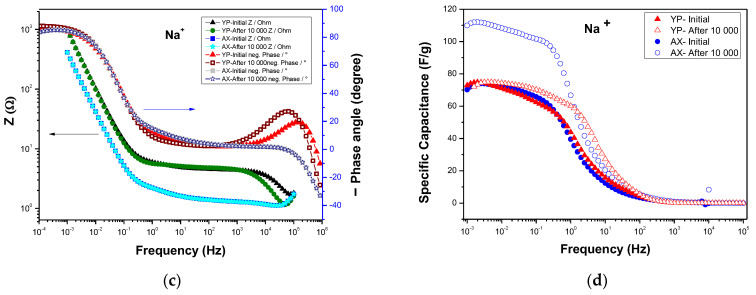
EIS of SC with AX and YP-50F and Na^+^– exchange Aquivion membrane before and after 10,000 cycles: (**a**) Nyquist plots (the inset shows the high frequency region), (**b**) cell resistance as a function of the frequency, (**c**) Bode plots, and (**d**) specific capacitance as a function of the frequency.

**Figure 10:**
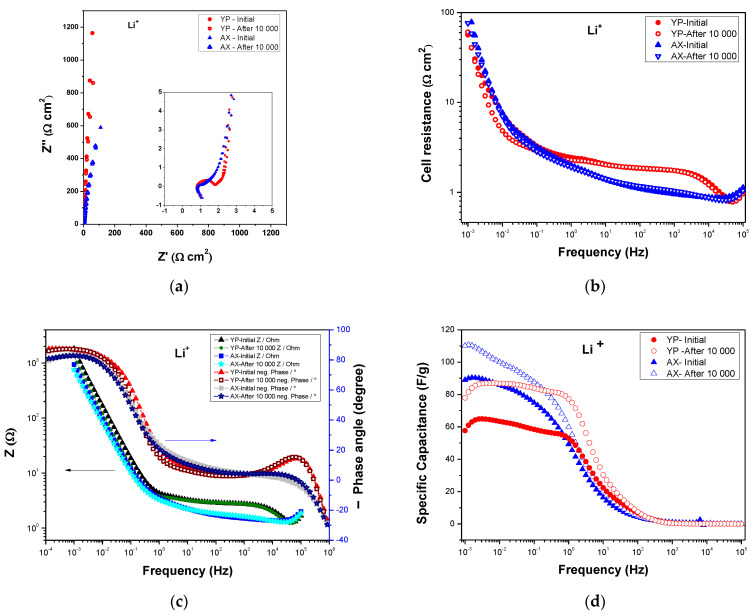
EIS of SC with AX and YP-50F and Li^+^– exchange Aquivion membrane before and after 10,000 cycles: (**a**) Nyquist plots (the inset shows the high frequency region), (**b**) cell resistance as a function of the frequency, (**c**) Bode plots, and (**d**) specific capacitance as a function of the frequency.

Furthermore, electrochemical impedance spectroscopy (EIS) was also carried out to elucidate the impedance features of various supercapacitors at the beginning of the tests and after 10,000 cycles. Nyquist plots for the different capacitors are presented in Figure 9a and Figure 10a. The internal resistance values of the resultant electrolyte membranes (R_s_, the lower Z’ value with imaginary impedance (Z″) > 0) demonstrate high Li^+^ and Na^+^ ions conductivities, with values of 7.9 and 6.8 mScm^−1^ for Na^+^– form and Li^+^–form Aquivion electrolyte membranes, respectively. These values of ion conductivity (e.g., Na^+^– and Li^+^–form) for solid electrolytes are lower than the proton conductivity (H^+^ conductivity) of the Aquivion membrane [35] at room temperature, ranging from 120–160 mScm^−1^ [35,36]. From the Nyquist plots, it is also noted that supercapacitors with YP-50 show a semicircle with a different radius due to an additional charge transfer resistance (R_ct_) that probably arises from secondary resistance for some faradic process occurring in the electrodes or due to electrode/current–collector interface resistances [36,37]. The first possibility in these spectra seems to be less likely because they are not present for all SCs with AX, even after tens of thousands of cycles. This enhanced behavior is confirmed in Figure 9d and Figure 10d, which show the increases in capacitance as the frequency decreases for both capacitors using Na^+^– and Li^+^–form Aquivion electrolyte membranes. However, in these graphs (Figure 9 and Figure 10), it is shown that the specific capacitance also increases at the end of the stability cycles, which is not an obvious finding, but it is probable that during the progression of the stability tests, an increased penetration and then wetting of ions (i.e., Na^+^, Li^+^) in the narrow pores may occur slowly with the help of hydrophilic carbon oxygen groups, as clearly shown in Figure 9b and Figure 10b. This latter possibility is highlighted as the resistance decreased as the stability tests proceed, and their values are lower after 10,000 cycles for the same capacitor, confirming this explanation.

A further very excellent result shown by the EIS analysis is well evidenced from the trend of phase shifts (phase angle), which are always ≥ to −83 degrees (i.e., (−) 83–88°) for all capacitors, and, in particular, the phase angles are even higher at the final stability tests, as can be seen in the Bode plots in Figure 9c and Figure 10c.

Phase shift values greater than −83° further support the fact that in these supercapacitors, there is no evidence of strong faradic reactions [38] as one might think from the semicircles in the Nyquist plot insets in Figure 9a and Figure 10a. Therefore, it is demonstrated that the appeared semi-circles (in insets in Figure 9a and Figure 10a) are to assign at interfacial electrical contact resistances.

**Figure 11:**
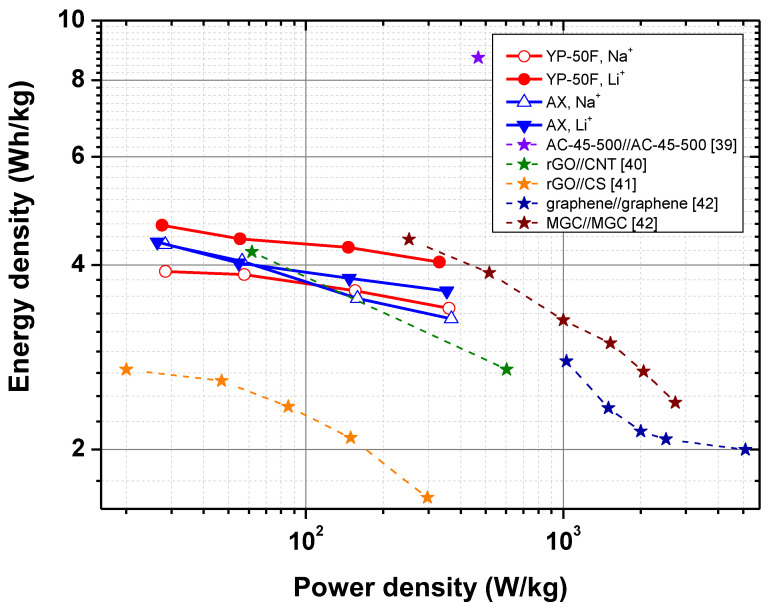
Energy density versus power density (Ragone plot) for SCs with AX and YP-50F and Na^+^– ^+^ and Li^+^–exchange Aquivion membrane. Results reported in the literature [39,40,41,42] are presented for comparison.

The electrochemical characteristics of the studied carbons were evaluated with Ragone plots for all supercapacitor cells in both electrolytes (Figure 11). The data in the figure show that YP-50F in Li^+^–form Aquivion electrolyte provides a specific energy density of 4.5 Wh kg^−1^ at a power of 50 Wkg^−1^ and maintains a relatively constant value of 4.0 Wh kg^−1^ even at a power density of 330 Wkg^−1^. In comparison, AX exhibits an energy density of 3.6 Wh kg^−1^ at a power density of 330 Wkg^−1^ when applied as an electrode in a Li_2_SO_4_ supercapacitor. The energy density of the supercapacitor using the Na^+^– form Aquivion membrane is lower. Although the values obtained are relatively low, they are higher compared to most of the SCs shown in Figure 11. Only the cell containing a carbon xerogel monolith shows a significantly higher power density value, which is attributed to the interconnected super macropore structure of the electrode material [39].

## 3. Conclusions

A carbon xerogel with designed and controlled properties was synthesized using microwave sol-gel methodology and investigated as an electrode material in the development of solid-state symmetric supercapacitors. The studies were conducted using Na^+^– and Li^+^–form Aquivion electrolyte membranes as the solid electrolyte, and a commercial activated carbon YP-50F was used for comparison.

The results of the study show that carbon xerogel (i.e., AX) and YP-50F exhibit good electrochemical performance, with low resistance and excellent cycling stability up to 10,000 charge–discharge cycles, and can be considered as suitable electrode materials for the development of solid-state supercapacitors.

Furthermore, it has been shown that the devices studied can operate with a suitable voltage window of 1.2 V and exhibit a specific capacitance between 105 and 110 F g^−1^ at 0.2 A g^−1^, which corresponds to an energy density of 4–4.5 Wh kg^−1^ at 300 W kg^−1^. These good electrochemical performance values can be attributed to the following reasons: (1) the good textural characteristics of carbon xerogel (i.e., AX); (2) the design of the cell configuration using Na^+^– and Li^+^– exchange Aquivion electrolyte membranes, which provide high ionic conductivities, as well as high electrochemical utilization and a tight seal after being sealed in the cells; and (3) the polymer electrolyte can prevent the loss of the sulfate electrolyte, which offers the possibility of assembling it into potential foldable devices.

## 4. Experimental Section

### 4.1. Synthesis of Carbon Xerogels

Sol-gel methodology was employed to obtain a synthetic carbon with very well controlled physicochemical properties. Resorcinol C_6_H_6_O_2_ (VWR Chemicals, AnalaR NORMA, Japan) and formaldehyde CH_2_O (Merck, Germany, 37% aqueous) were used as precursor monomers and they were dissolved in water that was used as the reaction media. The initial pH of the precursor mixture was around 3 and it was raised to 6.5 with NaOH in order to promote the polymerization reaction and to obtain a polymeric structure with a mean pore size ca. 10 nm, according to previous works [43,44]. To promote the sol-gel process, it is necessary to heat the mixture to a temperature always below 100 °C. In this case, the precursors were heated at 85 °C by means of microwaves. This particular manner of heating is a very simple, safe and quick method to heat aqueous samples, and in this case, it provides great advantages such as the short process time (i.e., 5 h) and the use of just one single device for the whole process (i.e., nucleation, crosslinking, curing, and drying the gel) in comparison with the traditional methodologies. The temperature in the microwave was controlled with a thermocouple introduced in the precursor mixture and connected to a PID controller that adjusted the power of the magnetron and the pulses to maintain a constant operating temperature (85 °C). After 5 h, the solid and dried polymer obtained underwent a post-synthesis treatment at 1000 °C under CO_2_ flow. This treatment produces two types of changes in the polymer: (i) a devolatilization process because of the heat treatment up to 1000 °C, where all labile functional groups (i.e., mainly oxygen groups) are removed and the sample elemental composition is concentrated in carbon; and (ii) an activation process due to the presence of CO_2_ as a reactant, where defects are produced in the carbon structure due to the partial gasification of the carbon structure with the oxidant reactant. These defects are mainly micropores, and they are created inside the nodules of the polymer, whilst the feeder pores of 10 nm created during the sol-gel process remain intact and they are located between nodules. Therefore, at the end of the process, an activated carbon xerogel (AX) is created with no impurities that is mainly composed of carbon (>95 wt%C), with feeder pores of 10 nm and a high volume of microporosity that would provide a high surface area.

### 4.2. Physicochemical Characterization of Carbon Xerogels

The carbon xerogel obtained was characterized using different techniques in order to have information about its chemical composition, morphology, and porous properties. The commercial activated carbon YP-50F from Kuraray Europe was also characterized for comparative purposes.

Elemental analysis and C, H and N contents of the samples were measured in a LECOCHNS-932 microanalyzer, while the O content was determined in a LECO-TF-900 device. Both samples present no impurities in their contents. The surface chemical composition may influence the interaction with electrolytes and, more specifically, the wetting behaviors of the samples. Therefore, the wetting angle was measured using an apparatus Force Tensiometer K100 of KRÜSS K100. The measurement consists of dropping 10 μL of the solution on the electrode made with the sample to study and observe the shape of the drop to obtain the value of the wetting angle. This measurement was performed 5 times in order to have an average value per electrode.

The morphology of the samples was observed using a scanning electronic microscope, SEM (Quanta FEG 650 microscope), while the porous properties were evaluated by recording N_2_ adsorption-desorption isotherms at 77 K (Micromeritics Tristar II). Samples were outgassed overnight at 120 °C before analysis. From these isotherms, the textural parameters, such as the specific surface area and micropore volume, can be obtained from the BET equation and NLDFT method. The total pore volume was obtained from the total amount of nitrogen adsorbed at the saturation point (i.e., P/P_0_ = 0.99).

### 4.3. Preparation of the Carbon Xerogel Electrodes

The composition of the prepared electrodes was 80 wt% of the activated carbon xerogel, 10 wt% poly-(vinylidene fluoride-co-hexafluoropropylene), and 10 wt% graphite fibers. The electrodes with activated carbon YP-50F were prepared with the same procedure and used for comparison.

The electrodes were prepared using a casting technique. Poly-(vinylidene fluoride-co-hexafluoropropylene) in grain form was dissolved in N,N-dimethylacetamide (3% solution) before use. For good homogenization of the materials, they were mixed and stirred for 15 min. A layer with a thickness of 500 μm formed on a glass plate using Film Applicator Elcometer 4340 and was dried at 40 °C for 5 h and 12 h at 70 °C, then separated using distilled water and finally dried at 120 °C for 1 h.

### 4.4. Activation of the Polymer Electrolyte Membrane and Electrochemical Characterization

As the electrolyte and separator, the Aquivion^®^E87-05S membrane with an equivalent weight of 870 g mol^−1^ and a thickness of 50 µm was used, which was purchased from Solvay. The membrane was activated to the Na^+^– and Li^+^–form by immersion in 1 M Na_2_SO_4_ and 1 M Li_2_SO_4_ solutions, respectively, for 24 h at room temperature before being assembled in the cell.

The activated Aquivion membrane with an area of 0.79 cm^2^ and the carbon electrodes with an area of 0.64 cm^2^ were mounted in a two-electrode coin Swagelok-type cell.

Electrochemical measurements of supercapacitors were performed using cyclic voltammetry (CV) at different scan rates that ranged from 10 to 50 mV s^−1^ in a voltage range of 0.05–1.2 V using a Multi PalmSens system (Model 4, Amsterdam, The Netherlands). The electrochemical impedance spectroscopy (EIS) measurements were performed using the same apparatus at frequencies ranging from a 10 MHz to 1 mHz. The galvanostatic charge–discharge measurements were performed using an Arbin Instrument System BT-2000 in the range between 0.05 and 1.2 V. The test program was executed at a constant current load of 100 to 1000 mAg^−1^ for 100 cycles per step. The cells were subjected to a long-term cycling tests at a current rate of 200 mAg^−1^ for 10,000 charge–discharge cycles. The specific capacitance, Cs, obtained from cyclic voltammetry was calculated as follows:

Cs = [4(I/(dV/dt))/m](1)

where I is the current, dV/dt is the voltage scan rate, and m is the mass of the active carbon material.

The following equation was used to calculate the specific capacitance (Fg^−1^) from the charge/discharge curves:

C = [4(I × ∆t)/(m × ∆V)](2)

where I (A), Δt (s), m (g) and ΔV (V) indicate the discharge current, discharge time, mass of the active material, and voltage window, respectively.

On the basis of the specific discharge capacitance, the energy density E (Whkg^−1^) and power density P (Wkg^−1^) were calculated using Equations (3) and (4):

E = C ΔV^2^/7.2 (3)

P = 3600 E/t(4)

## Figures and Tables

**Table 1:** Composition of the carbon xerogels and carbon YP-50F studied.

Sample	C (wt%)	H (wt%)	O (wt%)	N (wt%)	S (wt%)
AX	96.3	0.7	3.0	-	-
YP-50F	97.6	0.3	2.1	-	-

**Table 2:** Main porosity parameters for the carbon xerogel AX and the commercial carbon YP-50F obtained from N_2_ adsorption: specific surface area (S_BET_), external surface area (S_ext_), micropore volume (V_micro_), and total pore volume (V_t_).

Sample	S_BET,_ m^2^ g^−1^	S_ext,_ m^2^ g^−1^	V_micro,_ cm^3^ g^−1^	Vt, cm^3^ g^−1^
AX	1492	234	0.59	0.85
YP-50F	1756	157	0.62	0.80

**Table 3:** Coulombic efficiency of symmetric supercapacitors with AX and YP-50F using Na^+^– and Li^+^–form Aquivion membranes. Data after 1, 1000, 5000 and 10,000 GCD cycles are shown.

Coulombic Efficiency, %	1st Cycle		After 1000 Cycles		After 5000 Cycles		After 10,000 Cycles	
	Na^+^	Li^+^	Na^+^	Li^+^	Na^+^	Li^+^	Na^+^	Li^+^
AX	96	82	99	97	99	97	99	97
YP-50F	94	95	99	98	99	98	99	98

## Data Availability

The data that support the findings of this study are available within the articles.
